# Otitis media outcomes of a combined 10-valent pneumococcal *Haemophilus influenzae* protein D conjugate vaccine and 13-valent pneumococcal conjugate vaccine schedule at 1-2-4-6 months: PREVIX_COMBO, a 3-arm randomised controlled trial

**DOI:** 10.1186/s12887-021-02552-z

**Published:** 2021-03-08

**Authors:** Amanda Jane Leach, Edward Kim Mulholland, Mathuram Santosham, Paul John Torzillo, Peter McIntyre, Heidi Smith-Vaughan, Nicole Wilson, Beth Arrowsmith, Jemima Beissbarth, Mark D. Chatfield, Victor M. Oguoma, Peter Stanley Morris

**Affiliations:** 1 Child Health Division, Menzies School of Heath Research, Casuarina, Northern Territory Australia; 2 Charles Darwin University, Casuarina Northern Territory, Australia; 3 Murdoch Children’s Research Institute, Department of Paediatrics, University of Melbourne, Melbourne, Australia; 4 London School of Hygiene and Tropical Medicine, London, UK; 5 Johns Hopkins Bloomberg School of Public Health, Baltimore, USA; 6 Prince Alfred Hospital, Sydney, NSW Australia; 7 University of Sydney, NSW, Australia; 8 National Centre for Immunization Research and Surveillance, Sydney, NSW Australia; 9 Centre for Health Services Research Faculty of Medicine, University of Queensland, Brisbane QLD, Australia; 10 Health Research Institute University of Canberra, Canberra, ACT Australia; 11 Department of Paediatrics , Royal Darwin Hospital, Darwin, Northern Territory Australia

**Keywords:** Aboriginal, Infant, Otitis media, Pneumococcal conjugate vaccines, Combination schedule, PCV13, PHiD-CV10, 3-arm randomised controlled trial, Head-to-head

## Abstract

**Background:**

Aboriginal children living in Australian remote communities are at high risk of early and persistent otitis media, hearing loss, and social disadvantage. *Streptococcus pneumoniae* and non-typeable *Haemophilus influenzae* (NTHi) are the primary pathogens. We compared otitis media outcomes in infants randomised to either a combination of Synflorix™ (PHiD-CV10, with protein D of NTHi) and Prevenar13™ (PCV13, with 3, 6A, and 19A), with recommended schedules for each vaccine alone. We previously reported superior broader overall immunogenicity of the combination schedule at 7 months, and early superiority of PHiD-CV10 compared to PCV13 at 4 months.

**Methods:**

In an open-label superiority trial, we randomised (1:1:1) Aboriginal infants at 28 to 38 days of age, to either Prevenar13™ (P) at 2–4-6 months (_PPP), Synflorix™ (S) at 2–4-6 months (_SSS), or Synflorix™ at 1–2-4 months plus Prevenar13™ at 6 months (SSSP). Ears were assessed using tympanometry at 1 and 2 months, combined with otoscopy at 4, 6, and 7 months. A worst ear diagnosis was made for each child visit according to a severity hierarchy of normal, otitis media with effusion (OME), acute otitis media without perforation (AOMwoP), AOM with perforation (AOMwiP), and chronic suppurative otitis media (CSOM).

**Results:**

Between September 2011 and September 2017, 425 infants were allocated to _PPP(143), _SSS(141) or SSSP(141). Ear assessments were successful in 96% scheduled visits. At 7 months prevalence of any OM was 91, 86, and 90% in the _PPP, _SSS, and SSSP groups, respectively. There were no significant differences in prevalence of any form of otitis media between vaccine groups at any age. Combined group prevalence of any OM was 43, 57, 82, 87, and 89% at 1, 2, 4, 6, and 7 months of age, respectively. Of 388 infants with ear assessments at 4, 6 and 7 months, 277 (71.4%) had OM that met criteria for specialist referral; rAOM, pOME, or CSOM.

**Conclusions:**

Despite superior broader overall immunogenicity of the combination schedule at 7 months, and early superiority of PHiD-CV10 compared to PCV13 at 4 months, there were no significant differences in prevalence of otitis media nor healthy ears throughout the first months of life.

**Trial registration:**

ACTRN12610000544077 registered 06/07/2010 and ClinicalTrials.govNCT01174849 registered 04/08/2010.

**Supplementary Information:**

The online version contains supplementary material available at 10.1186/s12887-021-02552-z.

## Background

A 2008 analysis of the global burden of acute and chronic suppurative otitis media (AOM and CSOM) estimated an overall CSOM incidence rate of 4.76 per thousand people, 22.6% cases in under five-year-old children. In the first year of life, the lowest CSOM incidence rate was 1.59 per thousand in High Income Asia Pacific, the highest was in Oceania (36 per thousand) [1]. Australian Aboriginal and non-Aboriginal data were included in Oceania, combined and weighted by population size. The original national study of otitis media diagnoses in Australian primary healthcare settings reported that Aboriginal and Torres Strait Islander children were significantly more likely to have severe OM, particularly “discharging ears” which was 40-fold higher [2]. In remote Northern Territory (NT) communities, longitudinal birth cohort studies conducted in the pre- and post- 7-valent pneumococcal conjugate vaccine (PCV7) eras and including a maternal pneumococcal polysaccharide vaccination trial all found early nasopharyngeal colonisation and concomitant onset of otitis media (OM) within weeks of birth [3–5]. OM generally persisted throughout the first year of life, including 12% having chronic suppurative otitis media (CSOM) at 12 months of age, and less than 10% having bilaterally normal middle ears [4]. A West Australian study conducted in the early years of the PCV7 era also found that prevalence of OM and hearing loss were around 40% during first year of life [6]. Recent analyses using data-linkage has provided evidence that Aboriginal children with a history of hearing loss on entering school are at increased risk of vulnerability on entering school, [7] poor school attendance [8] and performance, [9] and substantiated child maltreatment [10].

During surveillance studies in the NT, culture of ear discharge from 83 cases of acute otitis media with perforation (AOMwiP) or CSOM in PCV7-vaccinees detected non-typeable *Haemophilus influenzae* (NTHi) in 58% cases and PCV7-type pneumococci in 5%, compared to 55 and 17%, respectively in non-PCV historic controls [11].

Few early-intervention trials for prevention or treatment of OM have been conducted in this population. One randomised controlled trial reported that long term antibiotics compared to placebo increase the resolution of OME and prevented progression to tympanic membrane perforation (TMP), with no increase in bacterial resistance [12]. Whilst effective, long term adherence is difficult and prevention strategies are the preferred approach.

At commencement of this trial, clinical trials of the effect of PCV7 on all-cause AOM found 6% relative risk reduction (RRR) in low-risk infants, and a lack of effect in a small RCT in high-risk infants [13]. The RRR was around 20% for pneumococcal AOM and 10% for recurrent AOM. The effect of a pre-cursor to PHiD-CV10, PD-11Pn, on all-cause AOM was 34% in healthy infants [14]. In the Northern Territory the childhood vaccination schedule transitioned through three PCVs over the period 7-valent PCV7 (2001 to 2009), ten-valent pneumococcal non-typeable *Haemophilus influenzae* protein D conjugated vaccine, PHiD-CV10 (2009 to 2011), and 13-valent PCV13 (from 2011) (see Table 1 and below for PCV13 and PHiD-CV10 formulations). Cross sectional surveys conducted prior to and during this current trial identified significant but modest reductions in the prevalence of severe OM and a significant reduction in NTHi-culture-positive middle ear discharge in PHiD-CV10 vaccinees compared to the PCV7 vaccinees [15–18]. The Cochrane review of PCVs in the prevention of all-cause acute otitis media was updated during the course of this RCT. RCTs of new PCVs including PHiD-CV10 were underway (including this RCT) and therefore not able to be included [14]. Our RCT was the only study proposing to evaluate a combination of PCVs within the primary series.

**Table 1:** Schedule of enrolment, interventions, and assessments

		Study period				
	**allocation**	**post allocation**				
Study visit number	**1**	**1**	**2**	**3**	**4**	**5**
Age (months)	**1**	**1**	**2**	**4**	**6**	**7**
Eligibility screen	x					
Informed consent signed	x					
Randomisation	x					
**Interventions – Pneumococcal conjugate vaccines**						
Prevenar13 (_PPP)		_	P	P	P	
Synflorix (_SSS)		_	S	S	S	
COMBO (SSSP)		S	S	S	P	
Rotarix^R^			x	x		
Infanrix^R^ Hexa			x	x	x	
**Outcome assessments**						
Risk factor data and interviews						
Fixed e.g. sex, birthweight, gestational age, maternal education	x					
Not fixed e.g. household occupancy, smoke exposure, breastfeeding	x				x	
Blood draw (heel, finger prick, or venepuncture)			x*	x*		x
Ear assessment						
Tympanometry		x	x	x	x	x
Video otoscopy				x	x	x
Nasopharyngeal swab		x§	x	x	x	x
General health (skin, chest, nose, temp, weight, length) and medical record review		x	x	x	x	x

Note: Outcomes of all procedures will be published in separate reports

*blood draw occurs at either 2 months or 4 months of age (decided by a random process). § NP swab collection at one month of age commenced late 2014 (NT) or 2015 (WA). S is PHiD-CV10 (Synflorix™). P is PCV13 (Prevenar13™)

PCV13 contains serotypes 1, **3,** 4, 5, **6A**, 6B, 7F, 9 V, 14, 18C, **19A**, 19F, and 23F

PHiD-CV10 contains serotypes 1, 4, 5, 6B, 7F, 9 V, 14, 18C, 19F, 23F, and **protein D** of non-typeable *Haemophilus influenzae*

### Objectives and hypotheses

We proposed that infants at high risk of early mixed infections would benefit from both PHiD-CV10 (Synflorix™, S) and PCV13 (Prevenar13™, P) given in the primary course schedule. We designed a 3-arm randomised controlled trial comparing a novel 4-dose primary course schedule of PHiD-CV10 at 1, 2, and 4 months plus PCV13 at 6 months (SSSP group) with standard 2–4-6-month schedules of each vaccine (_PPP and _SSS groups). To date we have reported primary and secondary immunogenicity and safety outcomes; the SSSP schedule was safe, and from 2 months of age immunogenicity was superior overall [19]. This paper reports otitis media outcomes by vaccine group at 1, 2, 4, 6 and 7 months of age. We also use data combined from all groups to describe the dynamics and analyse risk factors of otitis media in the first months of life.

## Methods

Details of the trial protocol have been published [20]. Brief summaries are provided.

### Trial design

PREVIX_COMBO is an open-label parallel superiority 3-arm (1:1:1) trial.

### Setting

The trial took place in five remote Aboriginal and Torres Strait Islander communities in the Northern Territory and Western Australia [20]. The PREVIX_COMBO trial was approved by the relevant Human Research Ethics Committees [20].

**Nomenclature** used in this manuscript are: italics *P* and *S* indicate vaccine (*P*revenar13™ or *S*ynflorix™) received at the time point of interest, and to indicate the comparison of interest (i.e. _PPP, _SSS, and *S*SSP at 2 months, _*P*PP, _*S*SS, and *SS*SP at 4 months, _*PP*P, _*SS*S, and *SSS*P at 6 months, and _*PPP*, _*SSS*, and *SSSP* at 7 months).

### Participants

Parents and families were provided with information about the studies from pregnancy and provided written informed consent and assent (from mothers younger than 16 years of age) at infant age 28 to 38 days of age. *Inclusion criteria*: Infants 28 to 38 days of age with gestational age > 32 weeks, eligible for routine immunisations, first born if twins, and whose families intended to remain in one of the five participating communities until infant age 7 months [20].

### Procedures and interventions

Research nurses were trained in giving vaccines and in standardised ear and general health checks. See Table 1 for schedule of procedures. Infants were randomised using a computer-generated sequence stratified by community and allocated (1:1:1) to one of three vaccine schedules: _PPP, _SSS or SSSP. Details of vaccine formulations are given elsewhere [20]. Briefly, ten-valent pneumococcal non-typeable *Haemophilus influenzae* conjugated vaccine, PHiD-CV10 (Synflorix™) contains ten pneumococcal serotypes, most of which are conjugated to protein D of *H. influenzae.* 13-valent pneumococcal conjugate vaccine, PCV13 (Prevenar™) has three additional serotypes, 3, 6A, and 19A, and all are conjugated to CRM_197_.

### Relevant concomitant care

Throughout this study, the Australian Indigenous infant vaccination schedule was EngerixB™ at birth, Rotarix^R^ at 2–4 months, Infanrix^R^ Hexa, and (for non-study participants) Prevenar13 at 2–4-6 months. Study staff provided treatment or referral for all concomitant conditions according to local guidelines.

### Secondary outcomes: otitis media

Ear assessments were made at scheduled study visits at 1, 2, 4, 6, and 7 months by research nurses trained in paediatric video otoscopy and tympanometry. Diagnostic categories (see Table 2) were made according to national guidelines for otitis media in Aboriginal and Torres Strait Islander populations and managed according to these and local guidelines (Central Australian Rural Practitioners Association Standard Treatment Manual). Tympanometry-only was used at 1 and 2 months of age, and both otoscopy and tympanometry were used at all subsequent visits. At 1 and 2 months of age a diagnosis of either normal (type A tympanogram) or OM (type B (flat) tympanogram) was made. For each child and time point a worst ear diagnosis and laterality were allocated as this determines medical management. The hierarchy of increasing severity was normal, OME, AOMwoP, DP, AOMwiP, and CSOM. Dry perforations were detected on two occasions and have been excluded from analyses.

**Table 2:** Otitis media abbreviations and definitions [21].

OM	Otitis media	All forms of inflammation and infection of the middle ear.
OME	Otitis media with effusion, “glue ear”	Presence of fluid behind the tympanic membrane without any acute symptomsor signs of inflammation.
pOME	Persistent otitis media with effusion	OME for more than 3 months without any acute symptoms.
AOMwoP	Acute otitis media without perforation	The presence of fluid behind the tympanic membrane plus at least one of thefollowing: bulging tympanic membrane, red tympanic membrane, fever, earpain or irritability.
AOMwiP	Acute otitis media with perforation	Discharge of pus (otorrhoea) through a small perforation (hole, generally < 2% ofthe pars tensa) in the tympanic membrane within the last 2 weeks.
rAOM	Recurrent acute otitis media	The occurrence of 3 or more episodes of AOM in a 6-month period, or occurrenceof 4 or more episodes in the last 12 months.
CSOM	Chronic suppurative otitis media	Persistent discharge of pus (otorrhoea) through a perforation (hole) in the tympanicmembrane lasting 2 weeks or more and tympanic membrane perforation largeenough to allow penetration of topical antibiotics into the middle ear space(generally > 2% of the pars tensa).
DP	Dry perforation or inactive CSOM	Presence of a perforation (hole) in the tympanic membrane without any signs ofdischarge or fluid behind the tympanic membrane.
TTO	Tympanostomy Tube otorrhoea	Discharge of pus (otorrhoea) through tympanostomy tubes (or “grommets”) in situ.
supp OM	Suppurative otitis media	Combined AOMwoP, AOMwiP, or CSOM.
TMP	Tympanic membrane perforation	Combined AOMwiP, DP, or CSOM

### Statistical analysis

The study sample size was estimated for analysis of immunogenicity. With this sample size we estimate 55% power to detect a doubling in the proportion of children free from OM at 7 months in the SSSP group (20%) compared to either _SSS or _PPP groups (10%), and 70% power when compared to _PPP and _SSS groups combined (10%). Vaccine group comparisons were tested with Fisher’s exact test for the proportion of infants with each diagnosis of otitis media at each timepoint; 95% confidence intervals (95%CI) were calculated. An intention to treat approach was used, including all available data. Longitudinal patterns of ear disease from 4 months of age are described from available data. We also conducted post-hoc logistic regression analyses of risk factors for any OM, suppurative OM, tympanic membrane perforation, or bilateral OM at 7 months of age. We included vaccine schedule, gender, community, mothers report of infant chest or ear infections, or runny nose, number of recent clinic visits for ear problems, family (mother and sibling) history of ‘runny ears’, number of other children the mother had, number of children under the age of 5 years in the household, breast and bottle feeding, maternal smoking during pregnancy, mother current smoker, smoker in the household, exposure to fire smoke. Risk factors with univariate *p* < 0.10 were included in multivariable logistic regression model for each OM outcome. All data were analysed using Stata/IC 15.1 [22].

### Data safety monitoring

The study was overseen by an independent Data Safety and Monitoring Board (iDSMB).

### Role of funding source

The funders had no role in design, collection, analysis, interpretation of data, writing the report or decision to submit for publication. As corresponding author, AJL had full access to all the data in the study and had final responsibility for the decision to submit for publication. AJL was not paid by any agency to write this article.

## Results

**Fig. 1:**
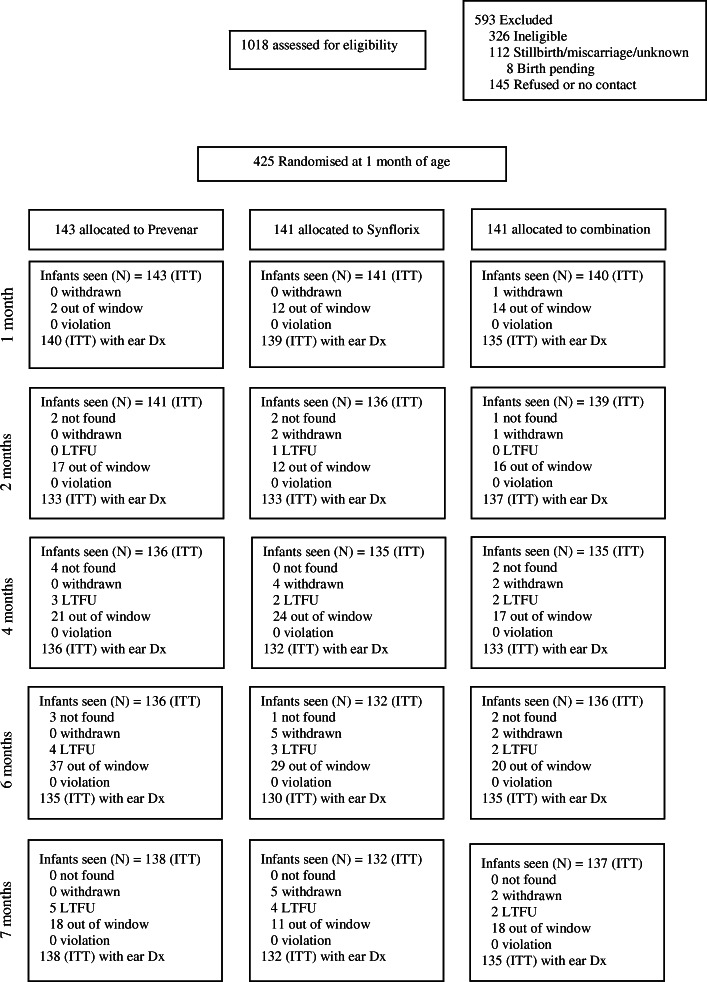
PARTICIPANT FLOW

Five communities commenced between September 2011 and August 2014. Of 1018 pregnancy notifications, 593 were excluded, 425 infants were randomised to _PPP (143), _SSS (141) or SSSP (141). 396 (93%) infants were randomised within the study window of 28 to 38 days of age (Fig. 1). Final randomisation of 425 infants occurred on 21st September 2017. Overall, infant birth characteristics were similar between groups (Table 3). Ear assessments were achieved in at least 90% infants across vaccine groups and time points (Fig. 1). Exclusion of protocol deviations or violations made no difference to our findings.

**Table 3:** Baseline Characteristics

Characteristics		Prevenar13 (_PPP)	Synflorix (_SSS)	Combo (**S**SSP)
		***N*** = 143	***N*** = 141	N = 141
Sex /gender	Male	77/143 (54%)	69/141 (49%)	70/141 (50%)
Gestational age at birth (Weeks)	Mean (SD)	38·4 (1·42)	38·4 (1·40)	38·1 (1·62)
Birth weight (kg)	Mean (SD)	3·15 (0·47)	3·19 (0·49)	3·07 (0·53)
Weight at randomisation (kg)	Mean (SD)	4·26 (0·54)	4·24 (0·61)	4·04 (0·68)
Age at randomisation (days)	Mean (SD)	33·1 (3·33)	32·5 (3·77)	32·5 (3·94)
Community	1 Wurrumiyanga	29/143 (20%)	32/141 (23%)	30/141 (21%)
	2 Wadeye	53/143 (37%)	50/141 (36%)	50/141 (36%)
	3 Kununurra	26/143 (18%)	25/141 (18%)	27/141 (19%)
	4 Alice Springs	7/143 (5%)	6/141 (4%)	6/141 (4%)
	5 Maningrida	28/143 (20%)	28/141 (20%)	28/141 (20%)
Have any of your other children had runny ears?	Yes	19/98 (19%)	22/97 (23%)	19/93 (20%)
Do you have other children?	Yes	120/123 (97.6%)	117/125 (93.6%)	114/121 (94.2%)
Are you breast feeding only?	Yes	98/117 (84%)	98/115 (85%)	93/114 (82%)
Are you breastfeeding?	Yes	120/124 (97%)	117/124 (94%)	116/121 (96%)
Are you bottle feeding only?	Yes	4/123 (3%)	6/120 (5%)	4/119 (3%)
Are you bottle feeding?	Yes	24/122 (20%)	26/125 (21%)	25/119 (21%)
Did you smoke when you were pregnant?	Yes	58/119 (49%)	60/124 (48%)	61/120 (51%)
Does anyone smoke at your house?	Yes	27/124 (22%)	31/125 (25%)	22/121 (18%)
Do you cook with or sit near a wood fire?	Yes	23/123 (19%)	22/125 (18%)	28/121 (23%)

### Baseline characteristics

There were no substantial vaccine group differences in key risk factors for OM (Table 3).

### Prevalence of bilaterally normal ears, by vaccine group and age

Prior to vaccination, at one month of age, tympanometry identified bilateral normal ears in 57, 59, and 54% infants in the _PPP, _SSS and SSSP groups, respectively. At 2 months, following a single dose of PHiD-CV10 in the *S*SSP group the prevalence of normal ears was 40, 48, and 40%, respectively. At 4 months, following a single dose of vaccine in the _*P*PP and _*S*SS groups and following two doses in the *SS*SP group, these figures were 20, 20, and 16%, respectively. At 6 months, following 2 doses in the _*PP*P and _*SS*S groups and three doses in the *SSS*P group, bilateral normal ears were detected in 10, 14 and 14%, and at 7 months 9, 14, and 10%. There were no statistically significant differences in the prevalence of bilaterally normal ears between vaccine groups at any age (Table 4).

**Table 4:** Comparisons of proportion of infants with a worst ear diagnosis* of each form of otitis media, by vaccine group and age (months)

Dx [worst*] Age (mo)	_PPP		_SSS		SSSP		SSSP vs _PPP			SSSP vs _SSS			_PPP vs _SSS		
	%	95%CI	%	95%CI	%	95%CI	%	95%CI	p	%	95%CI	p	%	95%CI	p
Normal															
1**	57	(49, 65)	59	(50, 67)	54	(46, 63)	−3	(−14, 9)	0.72	−5	(−16, 7)	0.47	−2	(− 13, 10)	0.81
2**	40	(32, 49)	48	(39, 57)	40	(32, 49)	0	(−12, 11)	1.0	−8	(−20, 4)	0.18	−8	(− 20, 4)	0.22
4	20	(14, 28)	20	(13, 28)	16	(10, 23)	−4	(−13, 5)	0.43	−4	(−13, 5)	0.43	0	(−9, 10)	1.0
6	10	(6, 17)	14	(8, 21)	14	(9, 21)	4	(− 4, 12)	0.46	0	(−8, 9)	1.0	−3	(−11, 4)	0.45
7	9	(5, 16)	14	(9, 22)	10	(5, 16)	0	(−7, 7)	1.0	−5	(−13, 3)	0.26	−5	(−13, 3)	0.26
OM															
1**	43	(35, 51)	41	(33, 50)	46	(37, 54)	3	(−9, 14)	0.72	5	(−7, 16)	0.47	2	(−10, 13)	0.81
2**	60	(51, 68)	52	(43, 61)	59	(50, 67)	−1	(−13, 10)	1.0	7	(−5, 19)	0.18	8	(−4, 20)	0.22
OME															
4	38	(30, 47)	48	(39, 57)	44	(36, 53)	6	(−6, 18)	0.43	−3	(−15, 9)	0.43	−9	(−21, 2)	1.0
6	34	(26, 43)	38	(29, 47)	40	(32, 49)	6	(−6, 17)	0.46	2	(−9, 14)	1.0	−4	(−15, 8)	0.45
7	30	(22, 38)	34	(26, 43)	34	(26, 42)	4	(−7, 15)	1.0	0	(−12, 11)	0.26	−4	(−15, 7)	0.26
AOMwoP															
4	39	(31, 48)	32	(24, 40)	37	(29, 46)	−2	(−14, 9)	0.80	5	(−6, 16)	0.44	7	(−4, 19)	0.25
6	43	(34, 52)	40	(32, 49)	39	(30, 47)	−4	(−16, 7)	0.54	− 1	(− 13, 10)	0.90	3	(−9, 15)	0.71
7	49	(40, 57)	42	(34, 51)	49	(41, 58)	1	(−11, 13)	1.0	7	(−5, 19)	0.27	6	(−6, 18)	0.33
AOMwiP															
4	3	(1, 7)	0	(0, 3)	2	(0, 6)	−1	(−4, 3)	1.0	2	(0, 5)	0.25	3	(0, 6)	0.12
6	10	(5, 16)	4	(1, 9)	5	(2, 10)	−4	(−11, 2)	0.24	1	(−4, 6)	0.77	6	(0, 12)	0.086
7	7	(3, 12)	6	(3, 12)	5	(2, 10)	−1	(−7, 4)	0.80	−1	(−6, 5)	0.80	0	(−5, 6)	1.0
CSOM															
4	0	(0, 3)	1	(0, 4)	1	(0, 4)	1	(−1, 2)	0.49	0	(−2, 2)	1.0	−1	(−2, 1)	0.49
6	3	(1, 7)	5	(2, 10)	2	(0, 6)	−1	(−5, 3)	1.0	−2	(−7, 2)	0.33	−2	(−6, 3)	0.53
7	6	(3, 11)	3	(1, 8)	2	(0, 6)	−4	(−8, 1)	0.22	−1	(−5, 3)	0.72	3	(−2, 8)	0.38
Any Supp^#^															
4	42	(34, 51)	33	(25, 41)	40	(31, 49)	−2	(−14, 10)	0.80	7	(−4, 19)	0.25	9	(−2, 21)	0.13
6	56	(47, 64)	48	(40, 57)	46	(37, 55)	−10	(−22, 2)	0.14	−3	(− 15, 9)	0.71	7	(−5, 19)	0.27
7	61	(52, 69)	52	(43, 60)	57	(48, 65)	−4	(−16, 7)	0.54	5	(−7, 17)	0.46	9	(−2, 21)	0.14
Any TMP^##^															
4	3	(1, 7)	1	(0, 4)	3	(1, 8)	0	(−4, 4)	1.0	2	(−1, 6)	0.37	2	(−1, 5)	0.37
6	13	(8, 19)	8	(4, 15)	7	(4, 13)	−5	(−12, 2)	0.22	−1	(−8, 5)	0.82	4	(−3, 11)	0.32
7	12	(7, 19)	9	(5, 15)	7	(4, 13)	−5	(−12, 2)	0.22	−2	(−8, 5)	0.66	3	(−4, 11)	0.44

95%CI: 95% Confidence Interval

*Dx worst is worst ear diagnosis according to a severity hierarchy of normal, OME, AOMwoP, AOMwiP, and CSOM

**At one and two months tympanometry was used to distinguish normal from not normal. We assumed not normal was OM, but these children may have had any form of OM

^#^Any Supp is suppurative OM which includes AOMwoP, AOMwiP, and CSOM

^##^Any TMP is tympanic membrane perforation which includes dry perforation, AOMwiP, and CSOM

S is PHiD-CV10 (Synflorix™). P is PCV13 (Prevenar13™)

### Prevalence of any OM or bilateral OM by age, and of each diagnostic category, by vaccine group and age

**Fig. 2:**
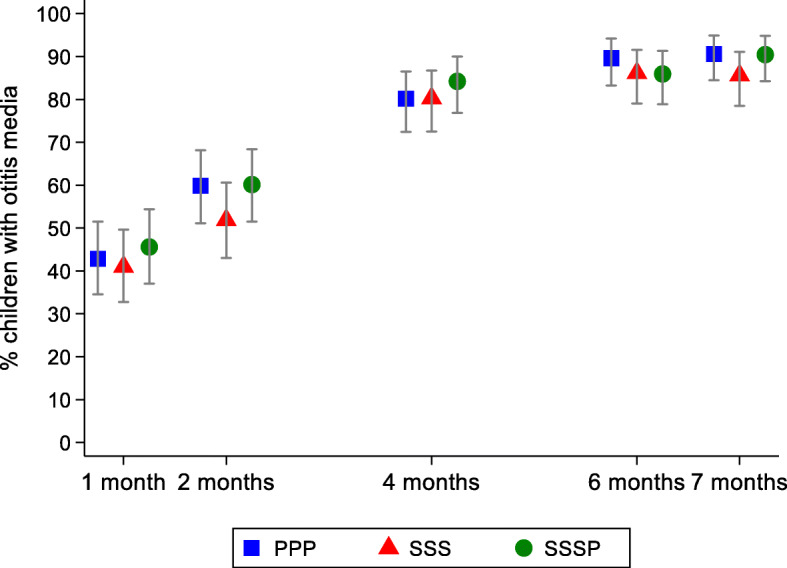
Prevalence (%, 95 confidence interval) of any otitis media, by age and vaccine group

At the primary endpoint of 7 months the prevalence of any OM was 91, 86, and 90% in the _*PPP*, _*SSS,* and *SSSP* groups. There were no statistically significant differences at any age in any OM (Fig. 2) or bilateral OM (Supplementary Table 1) between vaccine groups. For vaccine groups combined, any OM (in one or both ears) was detected in 43% infants at one month and 57% at 2 months, 82% at 4 months, 87% at 6 months, and 89% at 7 months. Bilateral OM was detected in 17, 35, 61, 74, and 76% at 1, 2, 4, 6, and 7 months, respectively (Supplementary Table 1).

**Fig. 3:**
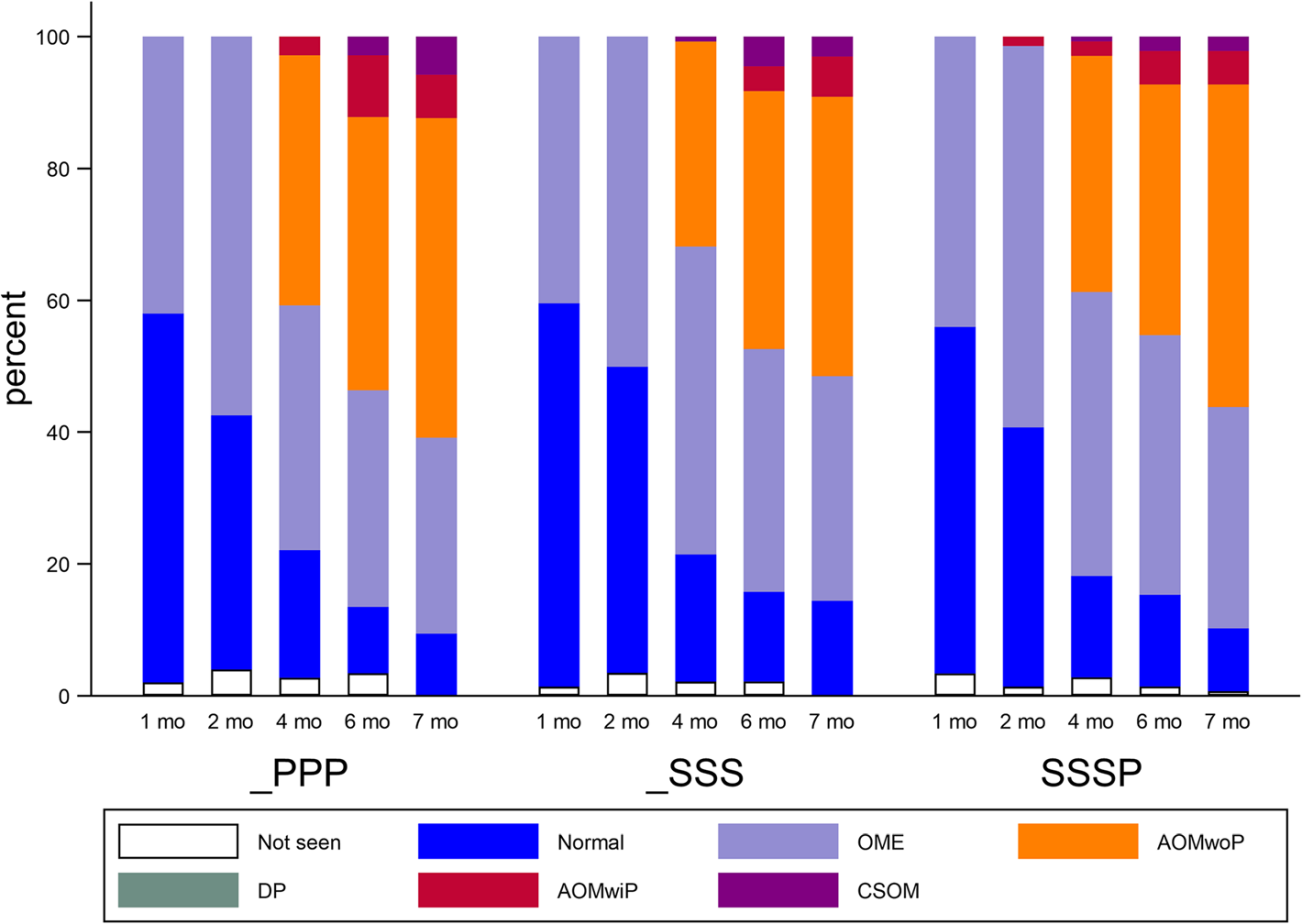
Proportion of infants (%) with a worst ear diagnosis* of each form of otitis media, by vaccine group and age (months)**

**Fig. 4:**
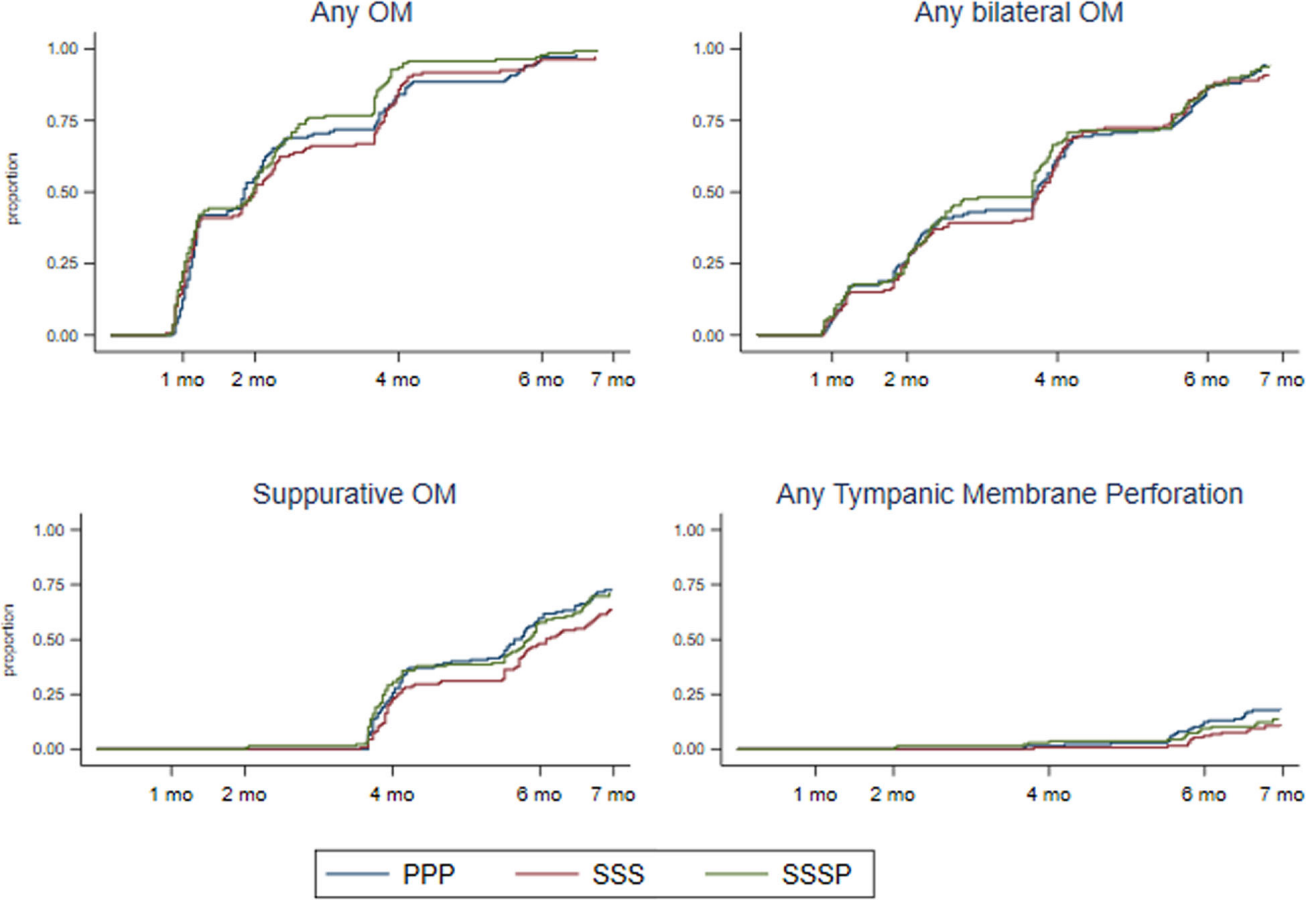
Cumulative proportion of infants with any OM, any bilateral OM, any suppurative OM^#^ or any tympanic membrane perforation^##^, by age and vaccine group

At 4, 6, and 7 months both tympanometry and otoscopy were performed, enabling diagnoses of OME, AOMwoP, AOMwiP, or CSOM (Table 2). The vaccine group prevalence of each type of OM at each time point by group (_PPP, _SSS, and SSSP) was: i) OME in 38, 48, and 44% infants at 4 months, 34, 38, and 40% at 6 months, and 30, 34, and 34% at 7 months; ii) AOMwoP in 39, 32 and 37% infants at 4 months. 43, 40, and 39% at 6 months, and 49, 42, and 49% at 7 months; iii) AOMwiP in 3, 0, and 2% infants at 4 months, 10, 4, and 5% at 6 months, and 7, 6, and 5% at 7 months; iv) CSOM in 0, 1, and 1% infants at 4 months, 3, 5, and 2% at 6 months, and 6, 3, and 2% at 7 months. Any suppurative OM (AOMwoP, AOMwiP, or CSOM) was the worst ear diagnosis in 42, 33, and 40% infants at 4 months, 56, 48, and 46% at 6 months, and 61, 52, and 57% at 7 months. Any tympanic membrane perforation (TMP, AOMwiP, DP, or CSOM) was the worst ear diagnosis in 3, 1, and 3% infants at 4 months, 13, 8, and 7% at 6 months, and 12, 9, and 7% at 7 months. There were no statistically significant differences in any OM diagnosis between vaccine groups at any age (Table 4, Figs. 3 and 4).

### Patterns of OM in 388 infants who had three successful consecutive ear assessments at 4, 6, and 7 months, vaccine groups combined

As there were no significant differences between vaccine groups, we combined data for further analyses by diagnosis and age. As we were unable to distinguish OME from AOM at 1 and 2 months, when tympanometry-only was used, the following analyses use diagnoses from infants seen at all three visits at 4, 6, and 7 months of age.

Overall, bilateral normal ears were rare and overall were found in just 14% diagnoses (Table 5). Around 37% infants had a diagnosis of OME, and 41% had AOMwoP. Tympanic membrane perforations were detected as early as 4 months (2.3%) and peaked at 7 months of age (10%). In this age group most perforations were AOMwiP (5% overall), and CSOM prevalence was almost 4% at age 7 months (Table 6).

**Table 5:** Prevalence (n, %) of OM at age 4, 6, and 7 months among 388 infants who had ear assessments at all three timepoints

Age	4 months	6 months	7 months	*Number of diagnoses*
*Number of children seen*	*388*	*388*	*388*	*1163*
Worst ear diagnosis				
Normal	74 (19.1%)	48 (12.4%)	41 (10.6%)	*163 (14.0%)*
OME	166 (42.8%)	144 (37.1%)	124 (32.0%)	*434 (37.3%)*
AOMwoP	139 (35.8%)	157 (40.5%)	184 (47.4%)	*480 (41.3%)*
AOMwiP	7 (1.8%)	25 (6.4%)	24 (6.2%)	*56 (4.8%)*
CSOM	2 (0.5%)	13 (3.4%)	15 (3.9%)	*30 (2.6%)*
Combined OM categories				
Supp OM^#^	148 (38.1%)	195 (50.3%)	223 (57.5%)	*566 (48.7%)*
TMP^##^	9 (2.3%)	38 (8.2%)	39 (10.0%)	*80 (6.9)*

#SuppOM, is suppurative OM which includes AOMwoP, AOMwiP, and CSOM

^##^TMP is tympanic membrane perforation which includes dry perforation, AOMwiP, and CSOM

**Table 6:** Proportion (n, %) of infants at 7 months who met referral criteria for persistent or severe OM, among 388 infants who had ear assessments at all three timepoints

Referral criteria	n (%) ***N*** = 388
Recurrent AOMwoP/AOMwiP (3 episodes within 6 months)	71 (18.3%)
Persistent OME (3 months)	47 (12.1%)
CSOM at least once	24 (6.2%)
Mixed OME/AOM (3 months)	135 (34.6%)
*Total referrals*	*277 (71.4%)*
**Other**	
Fluctuating (normal/OM)	110 (28.4%)
Normal (3 months)	4 (1.0%)

### Eligibility for specialist referrals

Guidelines recommend referral for specialist assessments of any child with recurrent AOM (rAOM) defined as three episodes in 6 months or four in 12 months, persistent OME (pOME) for at least 3 months, or any CSOM [21]. Recurrent AOMwoP or AOMwiP was detected in 71 (18%); pOME in 47 (12%), and CSOM in 24 (6%) infants. Thus 142 (37%) met standard criteria for referral. Importantly, an additional 135 (35%) infants had persistent mixed OME with AOMwiP or AOMwoP, making a total of 277 infants or a staggering referral rate of 71% (Table 6). Bilateral normal ears were detected at all three assessments in 4 infants (1%), at one or two assessments in 110 (28%) infants, and alternating with OME (45), AOMwoP (54), AOMwiP (9), or CSOM (2) (Table 6).

### Transitions from OM to normal middle ear status between each visit

Transitions from OM to no OM were very rare; 60 of 172 (35%) infants with OM at one month; 30 of 221 (14%) infants with OM at 2 months; 26 of 308 (8%) infants with OME or AOMwoP at 4 months; 27 of 307 (9%) infants with OME or AOMwoP at 6 months. Of 34 infants with AOMwiP at 4 or 6 months, two (6%) returned to normal at 7 months, and of 15 infants with CSOM at 4 or 6 months, none returned to normal ears at 7 months (Supplementary Table 2).

In univariate analyses we found no statistically significant risk factors for bilateral OM. For any OM, one community had an Odds Ratio (OR) of 0.37 (*p* = 0.03) compared to reference community. Statistically significant risk factors for suppurative OM and tympanic membrane perforations at 7 months were presence of prior ear discharge reported by the mother (OR 5.39 *p* < 0.001 and OR 55.6 p < 0.001, respectively), number of recent visits to the clinic for ear problems (OR 2.13 p = 0.03 and up to 8.71 p < 0.001, respectively), recent chest infections (OR 2.44 *p* = 0.01 for TMP only), recent runny nose (OR 2.29 p = 0.01 for TMP only), and mother having other children (OR up to 4.68 *p* < 0.01 for suppurative OM only). A multivariable analysis using a univariable approach (includes all predictors with *p* < 0.10) identified ear discharge (OR 4.98 *p* = 0.001) and number of children the mother had (OR up to 5.87 *p* = 0.006) as significant risk factors for suppurative OM. Ear discharge (OR 52.04 *p* < 0.0001) and number of visits to the clinic for ear problems (OR up to 5.45 *p* = 0.003) were significant risk factors for any TMP (Supplementary Table 3 for suppurative OM, any OM, and bilateral OM).

## Discussion

Despite indications of potential early and broadened immune protection from vaccine type pneumococci and NTHi, [19] our clinical findings clearly demonstrate a failure of this strategy to prevent early onset of OM in this population. We found no statistically significant differences in any OM diagnosis between vaccine groups at any age. Our study shows that PCVs do not directly or via herd effects, prevent early onset of all-cause OM during or one month after completing primary course schedules. Although tympanocentesis is not performed in Australia, nasopharyngeal carriage studies and culture of middle ear discharge during spontaneous perforation provide a close proxy for middle ear microbiology and confirm non-vaccine type pneumococci and NTHi as dominant pathogens in OM [11, 18]. We will report nasopharyngeal carriage outcomes at all timepoints for infants in this trial. In surveillance studies across the NT remote communities in 2013 (during this trial) we found that pneumococcal nasopharyngeal carriage in PCV13-vaccinated one-year old children was 77%, and the dominant serotypes were 16F (23%) and 15A (10%), followed by 23F, 11A, 35B, and 19F (each ~ 6%). Carriage of NTHi was 63% [18]. Respiratory viral infections may also contribute, although our previous work has shown only adenovirus is independently associated with AOM (~ 13% cases), whereas other viruses exacerbate bacterial OM through co-infection which increases bacterial load and risk of AOM [23].

In this high-risk population, and for infants at high-risk in other populations [24], prevention of early onset OM is critical as early onset is invariably followed by persistent and increasingly severe and intractable infections. Few vaccine studies have reported early otitis media outcomes, within the primary schedule. A 2016 systematic review of nasopharyngeal carriage following each primary dose of PCV7 or PHiD-CV10 found no significant difference between vaccinees and controls at 4 or 6 months, whereas at 7 months vaccinees had lower carriage of vaccine types, higher carriage of non-vaccine types [25]. No differences in overall pneumococcal or NTHi carriage were found at any timepoint. The latest update of the Cochrane review of pneumococcal vaccines for prevention of all-cause or pneumococcal AOM was to 29 March 2019 [14]. The review evaluated outcomes of early infancy schedules and administration in older children. The included studies had a minimum follow-up duration of 6 months and therefore likely measured outcomes after the booster dose. The effect of PHiD-CV10 varied from 6 to 15% RRR in healthy infants (non-significant) and there was no RCT evaluating otitis media outcomes of PCV13. The authors noted that in the absence of RCTs, data from population studies show further reductions in OM hospitalisations following PCV13 licensure, compared to PCV7 era [26]. The review also found that PCVs did not reduce all-cause AOM when administered in high risk infants, after early infancy, or in older children with a history of respiratory illness or frequent AOM. There was limited evidence that administration of PCVs in early infancy may reduce the risk of recurrent AOM. The authors suggested that the mechanism for this may be that PCVs prevent early vaccine-type AOM and thereby disrupt the progression and chronicity of subsequent all-cause AOM (non-vaccine serotype-AOM and NTHi-AOM) [27, 28]. Our study does not support this hypothesis. Although nasopharyngeal carriage of PCV13 serotypes has declined, we have seen replacement by non-PCV13 serotypes, and ongoing NTHi carriage.

The importance of preventing NTHi and non-PCV13-type early, persistent and severe OM in this populations is highlighted by our work. Our analysis of transitions in OM status during the first 6 months of life found less than 10% infants with OM had returned to normal healthy ears at subsequent visits. Where a diagnosis of bilaterally normal ears was made, this was transient, indicating the importance of regular and opportunistic ear assessments throughout the first months and years of life for these children. The National Guide for prevention of hearing loss in Aboriginal and Torres Strait Islander populations recommends timely vaccinations, surveillance, exclusive breast feeding, avoidance of smoke exposure, surgical interventions to reduce hearing impairment, chemoprophylaxis for high-risk children, and assessment of household overcrowding [29]. Many of these strategies prevent other infections and are broadly recommended. However, evidence for prevention of OM is weak or lacking, and where reported, effect size is generally small [21]. Our univariate and multivariable analyses of risk factors did not show statistically significant associations between OM and the classical risk factors, possibly due to high rates of OM with or without risk factor exposures. All infants in our study received appropriate management plans for their OM detected by research nurses at the time of vaccination, and according to otitis media guidelines for Aboriginal and Torres Strait Islander children, including education and guidance about detecting and managing ear and hearing problems, primary health care and specialist service referrals [21]. Very few infants received follow-up from their primary care providers, nor specialist services. The failure of these combined efforts to prevent and treat OM in these infants warrants a much greater local investment.

A limitation of our study was low sample size and limited ability to detect small but potentially important clinical differences between vaccines and schedules.

## Conclusion

Our study confirms that a multi-modal approach to otitis media prevention, detection, treatment, and support for hearing impaired children and their families will be required to tackle this crisis. Ongoing vaccine development to broaden pneumococcal serotype coverage and to elicit protective immune responses to NTHi is needed globally. New generation vaccines and schedules must be evaluated in high-risk populations. In Australia, strategies that address the social determinants of otitis media in Aboriginal and Torres Strait Islander children, particularly housing and access to quality health care can also contribute to elimination of childhood hearing impairment and social disadvantage. These too must be evaluated to ensure they are adequate, and that significant change is achieved.

## Supplementary Information

**Additional file 1 Supplementary Table 1:** Comparisons of proportion of infants with a worst ear diagnosis* of any otitis media or bilateral OM, by vaccine group and age (months).

**Additional file 2 Supplementary Table 2.** Proportion (n, %) of infants with transitions in OM status from 1 to 2, 2 to 4, 4 to 6, and 6 to 7 months of age, for infants with ear assessments at both timepoints (all vaccine groups combined).

**Additional file 3. Supplementary Table 3.** Univariable regression: odds ratios for suppurative OM, any OM, or bilateral OM at 7 months of age.

## Data Availability

The datasets generated and/or analysed during the current study are not publicly available due participant confidentiality but are available from the corresponding author on reasonable request.

## Abbreviations

AOMwiP: acute otitis media with perforation
AOMwoP: acute otitis media with perforation
CSOM: chronic suppurative otitis media
DP: Dry perforation
GSK: GlaxoSmithKline
NHMRC: National Health and Medical Research Council, Australia
NT: Northern Territory
NTHi: non-typeable *Haemophilus influenzae*
OM: otitis media
OME: otitis media with effusion
OR: Odds Ratio
P: 13-valent pneumococcal conjugate vaccine, Prevenar13™
PCV: pneumococcal conjugate vaccine
PCV7: 7-valent pneumococcal conjugate vaccine
PCV13: 13-valent pneumococcal conjugate vaccine, Prevenar13™
PHiD-CV10: 10-valent pneumococcal non-typeable *Haemophilus influenzae* conjugated vaccine, Synflorix™
pOME: persistent otitis media with effusion
PREVIX_COMBO: name of this RCT from PREVenar and synflorIX in COMBination
rAOM: recurrent acute otitis media
RCT: randomised controlled trial
RRR: relative risk reduction
S: 10-valent pneumococcal non-typeable *Haemophilus influenzae* conjugated vaccine, Synflorix™
TMP: tympanic membrane perforation
95%CI: 95% confidence interval
