# Supplementary material for: Otitis media outcomes of a combined 10-valent pneumococcal Haemophilus influenzae protein D conjugate vaccine and 13-valent pneumococcal conjugate vaccine schedule at 1-2-4-6 months: PREVIX_COMBO, a 3-arm randomised controlled trial
Source: BMC Pediatr. 2021 Mar 8;21:117. doi: 10.1186/s12887-021-02552-z (PMC7938290; doi:10.1186/s12887-021-02552-z)
Supplement: Supplementary file 1 — Additional file 1 Supplementary Table 1: Comparisons of proportion of infants with a worst ear diagnosis* of any otitis media or bilateral OM, by vaccine group and age (months). [file 12887_2021_2552_MOESM1_ESM.docx]

**Supplementary Table 1: Comparisons of proportion of infants with a worst ear diagnosis* of any otitis media or bilateral OM, by vaccine group and age (months)**

| Dx [worst*]  Age(mo) | Combined | _PPP |  | _SSS |  | SSSP |  | SSSP vs _PPP |  |  | SSSP vs _SSS |  |  | _PPP vs _SSS |  |  |
| --- | --- | --- | --- | --- | --- | --- | --- | --- | --- | --- | --- | --- | --- | --- | --- | --- |
|  |  | % | 95%CI | % | 95%CI | % | 95%CI | % | 95%CI | p | % | 95%CI | p | % | 95%CI | p |
| Any OM |  |  |  |  |  |  |  |  |  |  |  |  |  |  |  |  |
| 1 | 43% | 43 | (35, 51) | 41 | (33, 50) | 46 | (37, 54) | 3 | (-9, 14) | 0.72 | 5 | (-7, 16) | 0.47 | 2 | (-10, 13) | 0.81 |
| 2 | 57% | 60 | (51, 68) | 52 | (43, 61) | 60 | (51, 68) | 0 | (-11, 12) | 1.0 | 8 | (-4, 20) | 0.18 | 8 | (-4, 20) | 0.22 |
| 4 | 82% | 80 | (72, 86) | 80 | (72, 87) | 84 | (77, 90) | 4 | (-5, 13) | 0.43 | 4 | (-5, 13) | 0.43 | 0 | (-10, 9) | 1.0 |
| 6 | 87% | 90 | (83, 94) | 86 | (79, 92) | 86 | (79, 91) | -4 | (-12, 4) | 0.46 | 0 | (-9, 8) | 1.0 | 3 | (-4, 11) | 0.45 |
| 7 | 89% | 91 | (84, 95) | 86 | (78, 91) | 90 | (84, 95) | 0 | (-7, 7) | 1.0 | 5 | (-3, 13) | 0.26 | 5 | (-3, 13) | 0.26 |
| AnyOM bi |  |  |  |  |  |  |  |  |  |  |  |  |  |  |  |  |
| 1 | 17% | 18 | (12, 25) | 15 | (10, 22) | 18 | (12, 26) | 1 | (-9, 10) | 1.0 | 3 | (-6, 12) | 0.52 | 3 | (-6, 11) | 0.63 |
| 2 | 35% | 37 | (29, 46) | 31 | (23, 39) | 37 | (29, 46) | 0 | (-12, 11) | 1.0 | 6 | (-5, 17) | 0.31 | 6 | (-5, 18) | 0.3 |
| 4 | 61% | 61 | (52, 69) | 61 | (52, 70) | 60 | (51, 69) | -1 | (-13, 11) | 0.90 | -1 | (-13, 11) | 0.90 | 0 | (-12, 11) | 1.0 |
| 6 | 74% | 76 | (68, 83) | 75 | (67, 83) | 72 | (63, 79) | -4 | (-15, 6) | 0.49 | -4 | (-14, 7) | 0.58 | 1 | (-9, 11) | 0.89 |
| 7 | 76% | 80 | (73, 87) | 74 | (66, 81) | 73 | (65, 80) | -8 | (-18, 2) | 0.15 | -1 | (-12, 9) | 0.89 | 6 | (-4, 16) | 0.25 |

95%CI: 95% Confidence Interval

*Dx worst: Any OM is either ear with OME, AOMwoP, AOMwiP, or CSOM; AnyOM bi is both ears with any OM (bilaterally).

S is PHiD-CV10 (Synflorix^TM^). P is PCV13 (Prevenar13^TM^)
