# Supplementary material for: Otitis media outcomes of a combined 10-valent pneumococcal Haemophilus influenzae protein D conjugate vaccine and 13-valent pneumococcal conjugate vaccine schedule at 1-2-4-6 months: PREVIX_COMBO, a 3-arm randomised controlled trial
Source: BMC Pediatr. 2021 Mar 8;21:117. doi: 10.1186/s12887-021-02552-z (PMC7938290; doi:10.1186/s12887-021-02552-z)
Supplement: Supplementary file 2 — Additional file 2 Supplementary Table 2. Proportion (n, %) of infants with transitions in OM status from 1 to 2, 2 to 4, 4 to 6, and 6 to 7 months of age, for infants with ear assessments at both timepoints (all vaccine groups combined). [file 12887_2021_2552_MOESM2_ESM.docx]

**Supplementary Table 2. Proportion (n, %) of infants with transitions in OM status from 1 to 2, 2 to 4, 4 to 6, and 6 to 7 months of age, for infants with ear assessments at both timepoints (all vaccine groups combined).**

| First age | First diagnosis | **Follow-up diagnosis** | | | | | *Total* |
| --- | --- | --- | --- | --- | --- | --- | --- |
|  |  | **Normal**  **N (%)** | **OM**  **N (%)** |  | **AOMwiP****  **N (%)** |  |  |
|  |  | Follow-up age | | | | |  |
| 1 month* | 2 months | | | | | | |
|  | Normal | 113 (49.6) | 114 (50.0) |  | 1** (0.4) |  | *228 (57.0)* |
|  | OM | 60 (34.9) | 111 (64.5) |  | 1** (0.6) |  | *172 (43.0)* |
|  | *Total* | *173 (43.3)* | *225 (56.3)* |  | *2 (0.05)* |  | *400* |
|  | | **Follow-up diagnosis** | | | | |  |
|  |  | **Normal**  **N (%)** | **OME**  **N (%)** | **AOMwoP**  **N (%)** | **AOMwiP**  **N (%)** | **CSOM**  **N (%)** |  |
|  |  | Follow-up age | | | | |  |
| 2 months* | 4 months | | | | | | |
|  | Normal | 42 (25.3) | 68 (41.0) | 53 (31.9) | 2 (1.2) | 1 (0.6) | *166 (42.7)* |
|  | OM | 30 (13.6) | 100 (45.3) | 86 (38.9) | 5 (2.3) | 0 | *221 (56.8)* |
|  | AOMwiP** | 1**(0.3) | - | - | - | 1**(0.3) | *2 (0.5)* |
|  | *Total* | *73 (18.8)* | *168 (43.2)* | *139 (35.7)* | *7 (1.8)* | *2 (0.5)* | *389* |
| 4 months | 6 months | | | | | | |
|  | Normal | 22 (29.7) | 26 (35.1) | 21 (28.4) | 4 (5.4) | 1 (1.4) | *74 (18.9)* |
|  | OME | 18 (10.8) | 81(48.5) | 51 (30.5) | 11 (6.6) | 6 (3.6) | *167 (42.7)* |
|  | AOMwoP | 8 (5.7) | 37 (26.2) | 84 (59.6) | 9 (6.7) | 3 (2.1) | *141 (36.0)* |
|  | AOMwiP | 0 | 1 (14.3) | 2 (28.6) | 1 (14.3) | 2 (28.6) | *7 (1.7)* |
|  | CSOM | 0 | 0 | 1 (50) | 0 | 1 (0.3) | *2 (0.5)* |
|  | *Total* | *48 (12.3)* | *145 (37.1)* | *159 (40.7)* | *25 (6.4)* | *13 (3.3)* | *391* |
| 6 months | 7 months | | | | | | |
|  | Normal | 14 (27.5) | 18 (35.3) | 16 (33.3) | 2 (3.9) | 1 (2.0) | *51 (12.8)* |
|  | OME | 17 (11.6) | 72 (49.0) | 53 (35.9) | 3 (2.0) | 1 (0.7) | *147 (37.0)* |
|  | AOMwoP | 10 (6.3) | 32 (20.0) | 104 (66.2) | 9 (5.6) | 1 (0.6) | *160 (80.6)* |
|  | AOMwiP | 2 (8.0) | 3 (12.0) | 5 (20.8) | 8 (32.0) | 7 (28.0) | *25 (6.3)* |
|  | CSOM | 0 | 2 (15.4) | 4 (30.8) | 2 (15.4) | 5 (38.5) | *13 (3.3)* |
|  | *Total* | *43 (10.8)* | *123 (31.8)* | *188 (47.4)* | *24 (6.1)* | *15 (3.8)* | *397* |

* Diagnosis based on tympanometry-only (which indicates normal or ‘any OM’ (fluid behind the tympanic membrane), but does not distinguish between OM conditions

** Two infants with ear discharge observed by naked eye

Cells above the bold line represent transitions to same or worse diagnosis

Cells below the bold line represent transitions to improved diagnosis
