# Supplementary material for: Otitis media outcomes of a combined 10-valent pneumococcal Haemophilus influenzae protein D conjugate vaccine and 13-valent pneumococcal conjugate vaccine schedule at 1-2-4-6 months: PREVIX_COMBO, a 3-arm randomised controlled trial
Source: BMC Pediatr. 2021 Mar 8;21:117. doi: 10.1186/s12887-021-02552-z (PMC7938290; doi:10.1186/s12887-021-02552-z)
Supplement: Supplementary file 3 — Additional file 3. Supplementary Table 3. Univariable regression: odds ratios for suppurative OM, any OM, or bilateral OM at 7 months of age. [file 12887_2021_2552_MOESM3_ESM.docx]

Supplementary Table 3. Univariable regression: odds ratios for suppurative OM, any OM, or bilateral OM at 7 months of age

| Risk factor | N | Proportion (%) with  **suppurative OM** | Univariate Odds  Ratio | 95% CI | p | Proportion (%) with  **any OM** | Univariate Odds  Ratio | 95% CI | p | Proportion (%) with  **bilateral OM** | Univariate Odds  Ratio | 95% CI | p |
| --- | --- | --- | --- | --- | --- | --- | --- | --- | --- | --- | --- | --- | --- |
| Vaccine |  |  |  |  |  |  |  |  |  |  |  |  |  |
| _PPP | 138 | 60.9 |  |  |  | 90.6 |  |  |  | 80.4 |  |  |  |
| _SSS | 132 | 51.5 | 0.68 | 0.42, 1.11 | 0.12 | 85.6 | 0.62 | 0.29, 1.31 | 0.21 | 74.2 | 0.70 | 0.40, 1.24 | 0.23 |
| SSSP | 136 | 56.6 | 0.84 | 0.52, 1.36 | 0.48 | 90.4 | 0.98 | 0.44, 2.21 | 0.97 | 72.8 | 0.65 | 0.37, 1.15 | 0.14 |
| Gender |  |  |  |  |  |  |  |  |  |  |  |  |  |
| Male | 207 | 57.0 |  |  |  | 87.9 |  |  | . | 76.3 |  |  |  |
| Female | 199 | 55.8 | 0.95 | 0.64, 1.41 | 0.80 | 90.0 | 1.23 | 0.66, 2.29 | 0.52 | 75.4 | 0.95 | 0.60, 1.50 | 0.82 |
| Community |  |  |  |  |  |  |  |  |  |  |  |  |  |
| Wurrumiyanga | 88 | 55.7 |  |  |  | 90.9 |  |  |  | 78.4 |  |  |  |
| Wadeye | 152 | 62.5 | 1.33 | 0.78, 2.26 | 0.30 | 92.1 | 1.17 | 0.46, 2.97 | 0.75 | 79.0 | 1.03 | 0.54, 1.96 | 0.92 |
| Kununurra | 70 | 47.1 | 0.71 | 0.38, 1.33 | 0.29 | 78.6 | 0.37 | 0.15, 0.92 | **0.03** | 67.1 | 0.56 | 0.28, 1.15 | 0.11 |
| Alice Springs | 17 | 29.4 | 0.33 | 0.11, 1.02 | 0.05 | 82.4 | 0.47 | 0.11, 1.98 | 0.30 | 70.6 | 0.66 | 0.21, 2.11 | 0.48 |
| Maningrida | 79 | 59.5 | 1.17 | 0.63, 2.16 | 0.62 | 91.2 | 1.03 | 0.36, 2.98 | 0.96 | 76.0 | 0.87 | 0.42, 1.79 | 0.71 |
| Number of clinic visits for ears |  |  |  |  |  |  |  |  |  |  |  |  |  |
| none | 202 | 52.5 |  |  |  |  |  |  |  | 73.8 |  |  |  |
| 1 | 132 | 60.6 | 1.39 | 0.89, 2.17 | 0.14 | 66.2 | 1.10 | 0.56, 2.18 | 0.78 | 76.5 | 1.16 | 0.70, 1.93 | 0.57 |
| 2 | 47 | 70.2 | 2.13 | 1.08, 4.23 | **0.03** | 77.4 | 6.50 | 0.86, 49.22 | 0.07 | 80.9 | 1.50 | 0.68, 3.31 | 0.31 |
| >3 | 18 | 44.4 | 0.82 | 0.32, 2.09 | 0.67 | 86.0 | 0.53 | 0.16, 1.72 | 0.29 | 77.8 | 1.33 | 0.42, 4.20 | 0.62 |
| Has the baby had ear discharge |  |  |  |  |  |  |  |  |  |  |  |  |  |
| no | 349 | 52.2 |  |  |  | 88.0 |  |  |  | 74.2 |  |  |  |
| yes | 55 | 85.5 | 5.39 | 2.48, 11.74 | **<0.01** | 94.6 | 2.37 | 0.71, 7.93 | 0.16 | 85.5 | 2.04 | 0.93, 4.48 | 0.08 |
| Has the baby had any chest infection |  |  |  |  |  |  |  |  |  |  |  |  |  |
| no | 329 | 56.5 |  |  |  | 87.8 |  |  |  | 73.9 |  |  |  |
| yes | 75 | 57.3 | 1.03 | 0.62, 1.72 | 0.90 | 93.3 | 1.94 | 0.74, 5.09 | 0.18 | 84.0 | 1.86 | 0.96, 3.61 | 0.07 |
| Has the baby had any bad runny nose |  |  |  |  |  |  |  |  |  |  |  |  |  |
| no | 269 | 54.3 |  |  |  | 87.0 |  |  |  | 73.6 |  |  |  |
| yes | 135 | 61.5 | 1.34 | 0.88, 2.05 | 0.17 | 92.6 | 1.87 | 0.90, 3.90 | 0.10 | 80.0 | 1.43 | 0.87, 2.37 | 0.16 |
| Maternal history of runny ears |  |  |  |  |  |  |  |  |  |  |  |  |  |
| no | 283 | 55.5 |  |  |  | 88.7 |  |  |  | 74.9 |  |  |  |
| yes | 54 | 53.7 | 0.93 | 0.52, 1.67 | 0.81 | 83.3 | 0.64 | 0.29, 1.43 | 0.27 | 74.1 | 0.96 | 0.49, 1.86 | 0.90 |
| Do any of your other children have runny ears? |  |  |  |  |  |  |  |  |  |  |  |  |  |
| no | 262 | 54.2 |  |  |  | 87.4 |  |  |  | 72.5 |  |  |  |
| yes | 56 | 66.1 | 1.65 | 0.90, 3.01 | 0.11 | 91.1 | 1.47 | 0.55, 3.95 | 0.44 | 82.1 | 1.74 | 0.84, 3.64 | 0.14 |
| Number of other children |  |  |  |  |  |  |  |  |  |  |  |  |  |
| 0 | 18 | 27.8 |  |  |  | 88.9 |  |  |  | 77.8 |  |  |  |
| 1 | 125 | 60.8 | 4.03 | 1.35, 12.02 | **0.01** | 88.8 | 0.99 | 0.21, 4.77 | 0.99 | 76.0 | 0.90 | 0.28, 2.96 | 0.87 |
| 2 | 89 | 52.8 | 2.91 | 0.96, 8.85 | 0.06 | 84.3 | 0.67 | 0.14, 3.24 | 0.62 | 75.3 | 0.87 | 0.26, 2.92 | 0.82 |
| 3 | 56 | 64.3 | 4.68 | 1.46, 15.04 | **0.01** | 92.9 | 1.62 | 0.27, 9.71 | 0.59 | 82.1 | 1.31 | 0.36, 4.85 | 0.68 |
| 4 | 42 | 54.8 | 3.15 | 0.95, 10.42 | 0.06 | 85.7 | 0.75 | 0.14, 4.13 | 0.74 | 64.3 | 0.51 | 0.14, 1.85 | 0.31 |
| >5 | 18 | 61.1 | 3.57 | 0.90, 14.15 | 0.07 | 88.9 | 1.06 | 0.13, 8.47 | 0.95 | 72.2 | 0.80 | 0.18, 3.62 | 0.77 |
| Number of children under 5 years |  |  |  |  |  |  |  |  |  |  |  |  |  |
| 0 | 38 | 50.0 |  |  |  | 86.8 |  |  |  | 73.7 |  |  |  |
| 1 | 129 | 59.7 | 1.48 | 0.72, 3.06 | 0.29 | 89.1 | 1.24 | 0.42, 3.71 | 0.70 | 78.3 | 1.29 | 0.56, 2.97 | 0.55 |
| 2 | 114 | 55.3 | 1.24 | 0.59, 2.58 | 0.57 | 87.7 | 1.08 | 0.36, 3.23 | 0.89 | 71.9 | 0.92 | 0.40, 2.10 | 0.83 |
| 3 | 58 | 60.3 | 1.52 | 0.67, 3.47 | 0.32 | 87.9 | 1.10 | 0.32, 3.77 | 0.88 | 74.1 | 1.02 | 0.40, 2.60 | 0.96 |
| >4 | 24 | 46.2 | 0.71 | 0.25, 2.00 | 0.52 | 87.5 | 1.06 | 0.23, 4.91 | 0.94 | 79.2 | 1.36 | 0.40, 4.60 | 0.62 |
| Three or more children under 5 years |  |  |  |  |  |  |  |  |  |  |  |  |  |
| no | 281 | 56.6 |  |  |  | 88.3 |  |  |  | 75.1 |  |  |  |
| yes | 82 | 54.9 | 0.93 | 0.57, 1.53 | 0.78 | 87.8 | 0.96 | 0.45, 2.04 | 0.91 | 75.6 | 1.03 | 0.58, 1.82 | 0.92 |
| Currently breast feeding |  |  |  |  |  |  |  |  |  |  |  |  |  |
| no | 34 | 47.1 |  |  |  | 79.4 |  |  |  | 64.7 |  |  |  |
| yes | 332 | 57.5 | 1.52 | 0.75, 3.09 | 0.243 | 89.2 | 2.13 | 0.87, 5.25 | 0.10 | 76.2 | 1.75 | 0.83, 3.69 | 0.14 |
| Currently bottle feeding |  |  |  |  |  |  |  |  |  |  |  |  |  |
| no | 270 | 55.6 |  |  |  | 88.5 |  |  |  | 74.8 |  |  |  |
| yes | 93 | 58.1 | 1.11 | 0.69, 1.78 | 0.67 | 87.1 | 0.88 | 0.43, 1.79 | 0.71 | 75.3 | 1.02 | 0.59, 1.7 | 0.93 |
| Mother currently smokes |  |  |  |  |  |  |  |  |  |  |  |  |  |
| no | 143 | 54.5 |  |  |  | 88.8 |  |  |  | 79.0 |  |  |  |
| yes | 224 | 60.1 | 0.79 | 0.52, 1.20 | 0.29 | 88.0 | 0.92 | 0.48, 1.77 | 0.80 | 72.8 | 0.71 | 0.43, 1.17 | 0.18 |
| Mother smoked during pregnancy |  |  |  |  |  |  |  |  |  |  |  |  |  |
| no | 171 | 58.5 |  |  |  | 89.5 |  |  |  | 76.6 |  |  |  |
| yes | 166 | 54.2 | 0.84 | 0.55, 1.29 | 0.43 | 87.4 | 0.81 | 0.42, 1.59 | 0.54 | 73.5 | 0.85 | 0.52, 1.39 | 0.51 |
| Any smoker in the house |  |  |  |  |  |  |  |  |  |  |  |  |  |
| no | 287 | 58.5 |  |  |  | 87.8 |  |  |  | 73.9 |  |  |  |
| yes | 79 | 49.4 | 0.69 | 0.42, 1.14 | 0.15 | 89.9 | 1.23 | 0.55, 2.78 | 0.61 | 79.8 | 1.39 | 0.76, 2.56 | 0.29 |
| Cook or sit near a wood fire |  |  |  |  |  |  |  |  |  |  |  |  |  |
| no | 264 | 58.3 |  |  |  | 87.5 |  |  |  | 75.5 |  |  |  |
| yes | 102 | 52.0 | 0.77 | 0.49, 1.22 | 0.27 | 90.2 | 1.31 | 0.62, 2.78 | 0.47 | 75.0 | 1.03 | 0.60, 1.74 | 0.92 |
